# Flourishing in individuals undergoing maintenance hemodialysis in Shanghai: a Latent Profile Analysis

**DOI:** 10.3389/fpsyg.2026.1743933

**Published:** 2026-06-22

**Authors:** Yue-Qin Qian, Xiaoqing Zeng, Yiru Wang, Tianci Tong, Hongli Yan, Jing Wu, Jing Chu

**Affiliations:** 1 School of Nursing, Naval Medical University, Shanghai, China; 2 Department of Respiratory and Critical Care Medicine, The First Affiliated Hospital of Naval Medical University, Shanghai, China

**Keywords:** flourishing, influencing factors, Latent Profile Analysis, maintenance hemodialysis, Social Support Resource Theory

## Abstract

**Objective:**

This study aimed to reveal the profiles of the flourishing in undergoing maintenance hemodialysis (MHD) and to investigate influencing factors guided by the principles of Social Support Resource Theory.

**Methods:**

A total of 376 MHD patients were recruited between October and November 2022. The study used the PERMA Profiler of Chinese Version, Regulatory Emotional Self-Efficacy Scale, Perceived Social Support Scale, and Simplified Coping Style Questionnaire for data collection. Latent Profile Analysis was used to identify flourishing subgroups, and multiple regression analyses were used to explore the associations between latent profiles and sociodemographic and psychosocial characteristics.

**Results:**

Three distinct profiles were identified: the “Low Flourishing-Low Meaning” profile [*n* = 77 (20.48%)]; “Moderate Flourishing-Low Accomplishments” profile [*n* = 153 (40.69%)]; and the “High Flourishing-Low Engagement” profile [*n* = 146 (38.83%)]. Regression analysis revealed that coping style, social support, and regulatory emotional self-efficacy were significantly correlated with these subgroups.

**Conclusion:**

The flourishing levels of MHD patients are individualized. This study identified 3 distinct profiles of flourishing in MHD patients and found significant associations between these profiles. Our findings highlighted that targeted intervention should be developed based on the characteristics of each subgroup to improve their mental health and quality of life.

## 1 Introduction

Chronic kidney disease (CKD) has become a global health crisis, contributing significantly to both morbidity and mortality. According to the Global Burden of Disease Study 2023, CKD was responsible for an age-standardized rate of 769.2 disability-adjusted life-years (DALYs) per 100,000 population and 1.48 million deaths globally, with an age-standardized prevalence rate of 14.2% (95% UI: 13.4%–15.2%) (GBD 2023 Chronic Kidney Disease Collaborators, 2025). In China, the sixth China Chronic Disease and Risk Factor Surveillance found that the prevalence of CKD among adults aged 18 years or older is 8.2%, a figure expected to rise over the next decade (Wang L. et al., 2023). As kidney function deteriorates, many patients with CKD progress to end-stage renal disease (ESRD), requiring dialysis for survival. Hemodialysis is a renal replacement therapy for CKD that can remove metabolic waste and excess body water and rebalance electrolytes to sustain life (Flythe and Watnick, 2024). By 2025, according to the Chinese National Renal Data System (CNRDS), hemodialysis patients were estimated to account for 1,027,267 (Chen, 2025). The average age of patients undergoing maintenance hemodialysis (MHD) is steadily increasing, and MHD has evolved into a long-term treatment, often required for the remainder of a patient’s life.

However, while MHD prolongs survival, it is associated with numerous psychological challenges. Studies have shown that patients undergoing MHD frequently experience significant psychological distress, including anxiety, depression, and low self-esteem (Simões et al., 2019; Gadia et al., 2020; Chan et al., 2018; Musa et al., 2018). Research indicates that approximately 50% of hemodialysis patients report poor psychological conditions, with 32.5% experiencing severe depression and 54.1% suffering from severe anxiety; additionally, the co-occurrence of anxiety and depression is alarmingly high at 39.3% (Hou et al., 2014). These psychological issues significantly affect patients’ quality of life and their overall health outcomes, making it imperative to explore effective psychological interventions for this population.

Positive psychology, an emerging field focused on enhancing human well-being, offers valuable insights into improving the mental health of individuals facing chronic illness. The World Health Organization defines mental health as not merely the absence of mental disorders, but also the presence of positive well-being. Central to positive psychology is the concept of flourishing, which represents an optimal state of mental health. Flourishing includes experiencing positive emotions, engaging in meaningful activities, maintaining positive relationships, finding purpose in life, and achieving personal accomplishments (Agenor et al., 2017; Miao et al., 2013; Seligman, 2019). Seligman’s PERMA model of flourishing (Positive emotions, Engagement, Relationships, Meaning, and Accomplishments) underscores the multidimensional nature of well-being, advocating for a holistic approach to mental health that goes beyond the traditional focus on mental illness. As CKD patients, especially those on MHD, frequently experience compromised well-being, flourishing offers an alternative and comprehensive framework for understanding and improving their mental health.

Existing research on the psychological health of MHD patients has predominantly focused on negative mental health outcomes such as depression and anxiety (Ye et al., 2022; Xia et al., 2024; Jiang et al., 2023; Zhou et al., 2024). These studies often analyze psychological distress using total scores, which may overlook individual differences in psychological profiles. However, flourishing, as a positive psychological construct, is often underexplored in this context. Recent studies suggest that focusing solely on psychological deficits may fail to capture the complexity of patients’ mental health and well-being. Furthermore, traditional statistical analyses often fail to uncover the heterogeneity of psychological experiences within MHD patients, treating them as a homogenous group.

Latent Profile Analysis (LPA), a contemporary person-centered technique, offers a solution to this problem by identifying unobserved subgroups within a population based on the distribution of multiple variables (Howard and Hoffman, 2018). LPA has been shown to reveal nuanced psychological profiles, providing a more accurate understanding of the diverse ways in which MHD patients experience flourishing. This methodology can help identify distinct subgroups of patients with varying levels of flourishing, facilitating the development of more targeted psychological interventions.

The Social Support Resource Theory, grounded in the Conservation of Resources (COR) theory, posits that individuals draw upon both personal and social resources to cope with stress and promote well-being (Hobfoll et al., 1990). These resources include emotional, informational, and instrumental support, which play a crucial role in buffering the effects of stress and improving mental health outcomes. For MHD patients, social support has been identified as a critical factor in coping with the challenges of chronic illness, helping to mitigate feelings of isolation, anxiety, and depression (Zhou et al., 2024; Xie et al., 2023).

Despite the growing interest in social support, the Social Support Resource Theory has not yet been extensively applied to the study of flourishing in MHD patients. Understanding the role of social support resources–along with factors like emotional self-regulation efficacy and coping strategies–could provide valuable insights into how MHD patients can achieve a higher level of flourishing. Integrating this theory with the concept of flourishing offers a more comprehensive framework for addressing both the personal and social factors that contribute to well-being in this vulnerable population.

Therefore, based on the Theory of Resource Conservation, this study aims to investigate the characteristics of the level of flourishing in MHD from the perspective of positive psychology, analyze the differences in the level of flourishing in MHD, identify different subgroups of flourishing level, and analyze their status and characteristics, and explore the relationship between the level of flourishing and understanding social support, emotional self-regulation efficacy and coping styles. In addition, the potential effects of demography, disease-related factors, and psychosocial factors on the level of flourishing of MHD were discussed to provide the basis for formulating targeted intervention strategies. The study seeks to answer the following research questions:

1. How do emotional self-regulation efficacy, coping styles, and perceived social support interrelate to influence flourishing in MHD patients?

2. What latent profiles exist within MHD patients based on their levels of flourishing, and what are the characteristics of these profiles?

By addressing these questions, this study aims to provide a robust framework for enhancing the mental health and well-being of MHD patients, ultimately improving their quality of life and health outcomes.

## 2 Study design and participants

A multicenter cross-sectional study was conducted between October and November 2022, utilizing convenience sampling to recruit MHD patients from four hospitals in Shanghai. Eligibility criteria for participants include: (a) patient receiving MHD for end-stage renal disease; (b) Patients receiving hemodialysis for 3 months or longer; (c) The age of participants was more than 18 years; (d) conscious and oriented, enabling to understand scale content. Exclusion criteria include: (a) Recently undergone severe surgery; (b) Critically ill individuals; (c) cognitively impaired. A total of 376 patients were enrolled in this study. The detailed demographic information of participants is shown in Table 1.

**TABLE 1:** Demographic characteristics of MHD participants (*N* = 376).

Characteristics	*N* (%)
Gender	
Male	220 (58.5)
Female	156 (41.5)
Education	
Elementary or less	11 (2.9)
Middle school graduate	100 (26.6)
High school graduate	130 (34.6)
College or higher	135 (35.9)
Marital status	
Married	300 (79.8)
Unmarried	40 (10.6)
Widowed	17 (4.5)
Divorced	19 (5.1)
Primary caregiver	
Parent/child	66 (17.6)
Spouse	150 (39.9)
Self	151 (40.2)
Other	9 (2.4)
Employment	
Full-time job	77 (20.5)
part-time job	14 (3.7)
Retirement	241 (64.1)
Unemployment	44 (11.7)
Number of co-morbid disease	
None	73 (19.4)
1	141 (37.5)
2	99 (26.3)
3	41 (10.9)
≥4	22 (5.9)
Burden of medical expenses for disease	
Very light	48 (12.8)
Mild	100 (26.6)
Moderate	158 (42.0)
Severe	55 (14.6)
Very severe	15 (4.0)
Impact of disease on life	
Very light	11 (2.9)
Mild	56 (14.9)
Moderate	171 (45.5)
Severe	109 (29.0)
Very severe	29 (7.7)

## 3 Measures

### 3.1 Social-demographic information

Social-demographic information concerning gender, age, marital status, education level, occupation, and economic burden, duration of MHD, commodities, self-assessed severity, and life impact of MHD, the understanding of disease-related knowledge were collected with a self-designed questionnaire.

### 3.2 PERMA profiler of Chinese version (PP-C)

Flourishing was assessed with the PERMA scale developed by Butler and Kern (2016) and the Chinese version refined by our research group. The scale captures five subscales of flourishing: Positive emotions, Engagement, Relationships, Meaning, and Accomplishments. Participants rated each item on a ten-point scale. A total score was created by summing the scores from all five subscales, with higher scores indicative of a high level of well-being. The Cronbach’s α of the scale in this study was 0.895.

### 3.3 Regulatory emotional self-efficacy scale (RESS)

Emotional Self-Efficacy was measured with the 12-item Regulatory Emotional Self-Efficacy Scale (RESS) developed by Wen et al. (2009). The scale included three factors associated with emotional self-efficacy: Perceived self-efficacy in expressing positive affect, (POS, 4 items), Perceived self-efficacy in managing despondency (DES, 5 items), and Perceived self-efficacy in managing anger (ANG, 3 items). Each item is scored on a five-point scale, ranging from 1 to 5. The total scale score for this study ranges from 12 to 60. Higher scores indicate a greater level of emotional self-efficacy. The Cronbach’s α of the scale in this study was 0.872.

### 3.4 Perceived social support scale (PSSS)

Social support was collected using the 12-item scale of Perceived Social Support Scale (PSSS) (Xiao, 1995). Two subscales evaluate participants’ levels of family support and friends and others support. Participants rated each item on a seven-point scale. Total scores range from 12 to 84, with higher scores indicative of a greater perception of social support. The Cronbach’s α of the scale in this study was 0.875.

### 3.5 Simplified coping style questionnaire (SCSQ)

The SCSQ encompasses 20 items that evaluate positive and negative coping strategies, as referenced by Huang et al. (2021). Each item is scored on a four-point Likert scale (0–3), with higher scores indicating a greater reliance on the corresponding coping style. In this study, the Cronbach’s alpha for the entire questionnaire was 0.845, including 0.856 for positive coping and 0.630 for negative coping.

## 4 Data collection

Before initiating the survey, approval was granted by the management of the hospital’s hemodialysis unit. Participants received clear explanations of the study’s purpose and procedures, and informed consent was obtained before questionnaire distribution. Upon completion, the questionnaires were gathered immediately by the researcher, who checked for omissions or inaccuracies on the spot. When necessary, participants were promptly asked to verify and correct any errors. This research strictly complied with the ethical guidelines outlined in the Declaration of Helsinki and received formal approval from the Naval Medical University Ethics Review Committee.

## 5 Statistical analyses

All data were initially entered and managed using EpiData 3.0. IBM SPSS 26.0 was employed for descriptive statistics and multiple regression analyses. LPA was carried out in Mplus 8.3. The optimal model was determined based on lower values of the Akaike Information Criterion (AIC), Bayesian Information Criterion (BIC), and sample-adjusted BIC (aBIC) (Kim, 2014). An entropy value exceeding 0.8 indicated an acceptable model and significant *p*-values for LMRT and BLRT suggested that the K-class model outperformed the K-1-class model (Kim, 2014). Mean and standard deviation were used to summarize scale scores, while ANOVA and chi-square tests were used to examine differences across demographic variables. Disordered multi-classification analysis was conducted to identify factors influencing flourishing. A *p*-value of 0.05 was considered statistically significant.

## 6 Results

### 6.1 Model selection

Fit statistics for different latent profile models are shown in Table 2. Latent Profile Analysis was conducted based on the five dimensions of the Flourishing Scale, with models ranging from one to four latent profiles. The results showed that the AIC, BIC, and aBIC values progressively decreased as the number of model profiles increased. We chose the three-profile solution, as it provided lower AIC, BIC, and aBIC than the solutions with a smaller number of profiles, with significant LMRT and BLRT values (*P* < 0.05), and the ENtropy was at an ideal level, indicating a significant improvement in fit indexes compared to the K−1 solution (i.e., two-profile solution). The four-profile solution had slightly better-fit indexes in terms of AIC, BIC, aBIC, only with significant BLRT values. Therefore, the three-profile model was selected as the final solution for this study.

**TABLE 2:** Fit statistics for profile structure.

Model	AIC	BIC	aBIC	ENtropy	pLMR	pBLRT	Class probability
1	8284.97	8324.26	8292.54	–	–	–	–
2	7639.77	7702.65	7651.88	0.86	<0.001	<0.001	0.40, 0.60
**3**	**7389.60**	**7476.06**	**7406.26**	**0.87**	**<0.001**	**<0.001**	**0.20, 0.41, 0.38**
4	7279.66	7389.69	7300.85	0.87	0.1633	<0.001	0.16, 0.39, 0.08, 0.37

Numbers in bold indicate “best” fit. AIC, Akaike Information Criteria; BIC, Bayesian Information Criteria; aBIC, adjusted BIC; pLMR, *p*-value for Lo-Mendell-Rubin adjusted likelihood ratio test for K vs. K−1 profiles; pBLRT, *p*-value for Bootstrapped Likelihood Ratio Test.

### 6.2 Naming of three profiles

**FIGURE 1:**
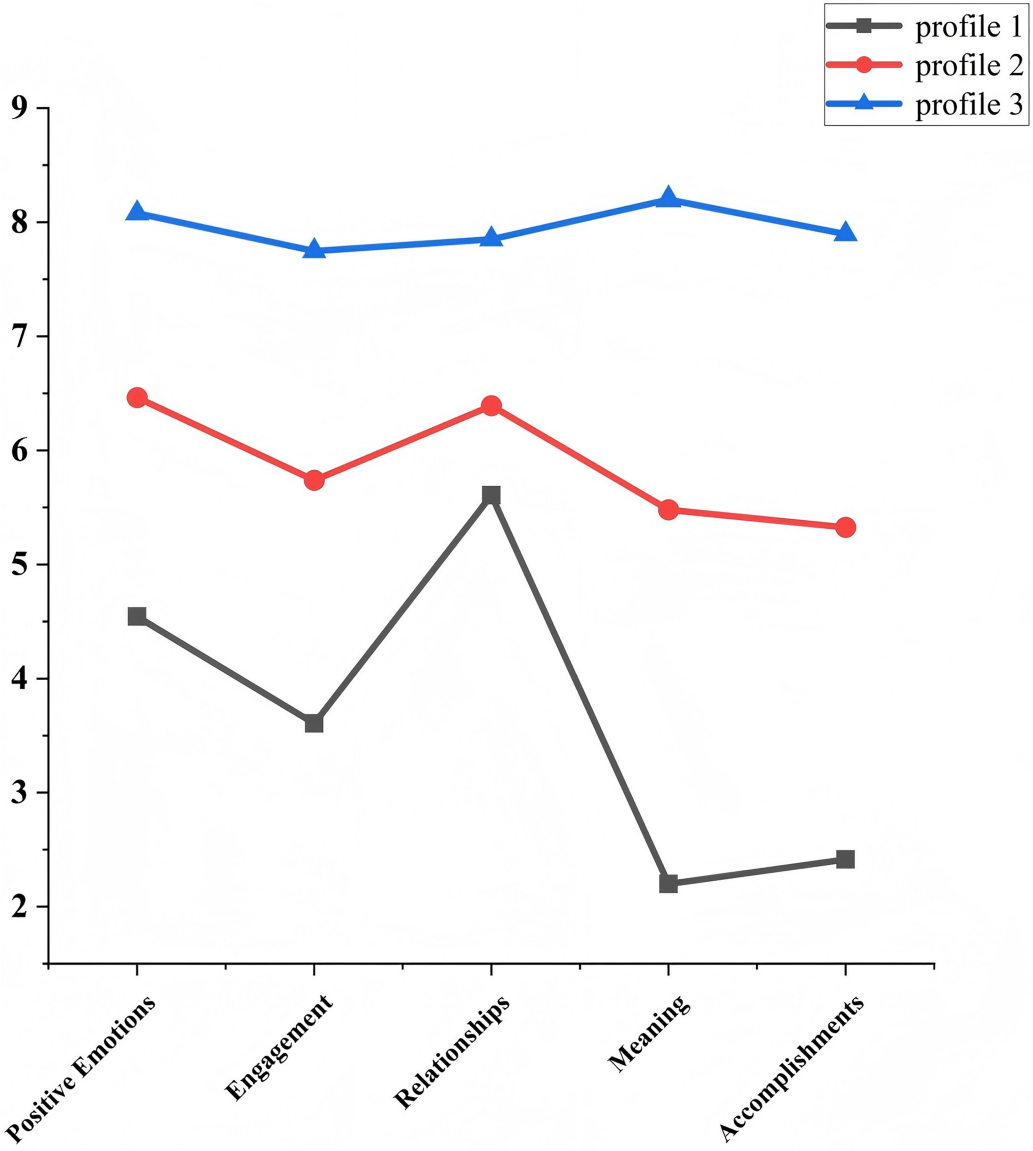
Latent profile of the flourshing. Profile 1, Low Flourishing-Low Meaning; Profile 2, Moderate Flourishing-Low Accomplishments; Profile 3, High Flourishing-Low Engagement.

The estimated means of the five dimensions of the Flourishing Scale are shown in Figure 1. Based on the explicit characteristics of each scale dimension, the profiles were named accordingly. We labeled the profile with the lowest Flourishing and the lowest score in the Meaning dimension (*M* = 2.17) as “Low Flourishing-Low Meaning” (20.48%). Individuals with moderate levels of flourishing and low accomplishments (*M* = 5.3) were labeled as Moderate Flourishing-Low Accomplishments (40.69%). We labeled the profile with the highest level of flourishing and lower engagement (38.83%) as High Flourishing-Low Engagement.

### 6.3 Influences of demographic variables for the three profiles

Comparisons of the three latent profiles of Flourishing among MHD patients revealed statistically significant differences in Education, Marital Status, Employment, Number of Co-morbid Diseases, and Impact of Disease on Life (*P* < 0.05). Detailed results are provided in Table 3.

**TABLE 3:** Influences of demographic variables on the three profiles.

Characteristics	Profile 1 (*N*)	Profile 2 (*N*)	Profile 3 (*N*)	*P*-value
Gender				0.286
Male	51	88	81	
Female	26	65	65	
Education				<0.001
Elementary or less	6	1	4	
Middle graduate	29	42	29	
High graduate	27	59	44	
College or higher	15	51	69	
Marital status				0.016
Married	58	120	122	
Unmarried	10	14	16	
Widowed	8	6	3	
Divorced	1	13	5	
Primary caregiver				0.060
Parent/child	15	30	21	
Spouse	32	64	54	
Self	25	58	68	
Other	5	1	3	
Employment				0.010
Full-time job	4	35	38	
part-time job	5	6	3	
Retirement	56	94	91	
Unemployment	12	18	14	
Number of co-morbid disease				0.003
None	11	28	34	
1	18	59	64	
2	24	42	33	
3	16	14	11	
≥4	8	10	4	
Impact of disease on life				0.009
Very light	1	6	4	
Mild	4	24	28	
Moderate	29	69	73	
Severe	34	43	32	
Very severe	9	11	9	

Profile 1, Low Flourishing-Low Meaning; Profile 2, Moderate Flourishing-Low Accomplishments; Profile 3, High Flourishing-Low Engagement.

### 6.4 Influences of psychological variables for the three profiles

**FIGURE 2:**
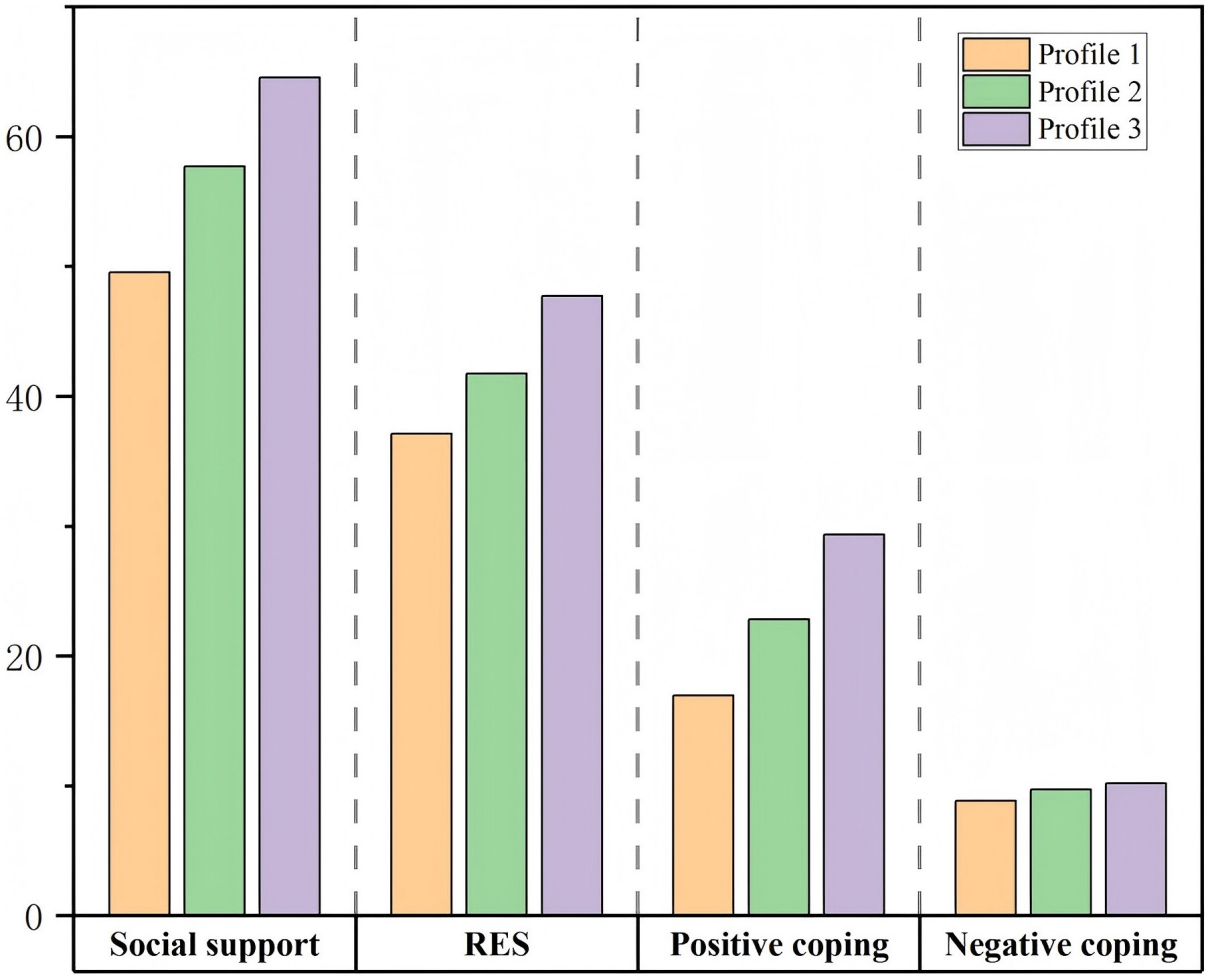
Conditional mean values of the three latent profiles on influence factors. RES, regulatory emotional self-efficacy.

Significant differences were observed among the three latent profiles of MHD patients in terms of Social Support, regulatory emotional self-efficacy, Coping Styles, and the scores for each dimension (*P* < 0.05). Detailed results are presented in Figure 2.

### 6.5 Factors associated with flourishing

Initially, parallelism tests were performed on each variable, and the results indicated a *p*-value of 0.017, suggesting that the parallelism assumption was not met. As a result, a multinomial logistic regression model was employed. The three latent profiles of Flourishing were set as the dependent variable, and variables that showed statistical significance in the univariate analysis were incorporated into the multinomial logistic regression model. The model demonstrated a statistically significant fit (χ^2^ = 277.037, *P* < 0.001). Significance analysis revealed that education, marital status, RES, social support, and coping style are significant factors influencing the different latent profiles of MHD patients (*P* < 0.05). The detailed results are shown in Table 4.

**TABLE 4:** Logistic regression analysis of three profiles.

Variables	Profile 1 compared to Profile 3				Profile 2 compared to Profile 3				Profile 1 compared to Profile 2			
	*B*	*P-*value	OR	95% CI	*B*	*P-*value	OR	95% CI	*B*	*P-*value	OR	95% CI
RES	−0.078	0.019	0.925	[0.866, 0.987]	−0.077	0.003	0.926	[0.880, 0.974]	0.001	0.957	1.001	[0.952, 1.053]
Social support	−0.069	0.001	0.933	[0.895, 0.973]	−0.025	0.102	0.976	[0.947, 1.005]	0.045	0.011	1.046	[1.010, 1.082]
Positive coping	−0.335	0.000	0.716	[0.656, 0.781]	−0.196	0.000	0.822	[0.771, 0.876]	0.138	0.000	1.148	[1.076, 1.226]
Negative coping	0.184	0.004	1.202	[1.060, 1.363]	0.106	0.010	1.112	[1.026, 1.205]	−0.078	0.173	0.925	[0.827, 1.035]
Education												
Elementary or less	1.313	0.304	3.716	[0.304, 45.443]	−0.968	0.454	0.380	[0.030, 4.789]	−2.281	0.077	0.102	[0.008, 1.285]
Middle graduate	0.623	0.300	1.865	[0.573, 6.068]	0.343	0.412	1.410	[0.620, 3.204]	−0.280	0.579	0.756	[0.281, 2.032]
High graduate	1.403	0.015	4.067	[1.312, 12.606]	0.967	0.007	2.629	[1.308, 5.284]	−0.436	0.393	0.646	[0.238, 1.759]
College or higher												
Marital status												
Married	1.102	0.444	3.010	[0.179, 50.459]	−1.594	0.030	0.203	[0.048, 0.856]	−2.696	0.030	0.067	[0.006, 0.773]
Unmarried	1.568	0.322	4.797	[0.215, 106.950]	−1.568	0.067	0.209	[0.039, 1.119]	−3.136	0.022	0.043	[0.003, 0.635]
Widowed	1.408	0.417	4.087	[0.136, 122.445]	−1.906	0.100	0.149	[0.015, 1.437]	−3.314	0.018	0.036	[0.002, 0.561]
Divorced												
Employment												
Full-time job	−1.107	0.205	0.330	[0.060, 1.832]	0.112	0.834	1.119	[0.392, 3.197]	1.220	0.116	3.386	[0.741, 15.469]
Part-time job	1.342	0.217	3.826	[0.454, 32.202]	0.520	0.563	1.682	[0.288, 9.822]	−0.822	0.342	0.440	[0.081, 2.392]
Retirement	0.781	0.287	2.185	[0.518, 9.211]	0.553	0.294	1.738	[0.619, 4.879]	−0.229	0.711	0.796	[0.237, 2.666]
Unemployment												
Number of co-morbid disease												
None	0.246	0.812	1.279	[0.169, 9.690]	−0.303	0.712	0.738	[0.147, 3.703]	−0.549	0.456	0.577	[0.136, 2.448]
1	−0.458	0.639	0.633	[0.094, 4.275]	−0.620	0.436	0.538	[0.113, 2.561]	−0.162	0.807	0.851	[0.233, 3.107]
2	0.033	0.973	1.034	[0.151, 7.076]	−0.397	0.625	0.672	[0.137, 3.299]	−0.430	0.515	0.650	[0.178, 2.372]
3	−0.329	0.757	0.719	[0.089, 5.804]	−1.176	0.199	0.309	[0.051, 1.856]	−0.847	0.234	0.429	[0.106, 1.731]
≥4												
Impact of disease on life												
Very light	−1.624	0.285	0.197	[0.010, 3.874]	−0.144	0.883	0.866	[0.127, 5.909]	1.480	0.264	4.394	[0.327, 59.002]
Mild	−1.812	0.077	0.163	[0.022, 1.215]	−0.249	0.724	0.779	[0.196, 3.105]	1.562	0.071	4.769	[0.875, 26.009]
Moderate	−1.457	0.080	0.233	[0.046, 1.191]	−0.584	0.367	0.558	[0.157, 1.982]	0.873	0.194	2.394	[0.641, 8.943]
Severe	0.745	0.378	2.107	[0.402, 11.040]	0.568	0.401	1.764	[0.469, 6.634]	−0.177	0.791	0.837	[0.226, 3.107]
Very severe												

RES, regulatory emotional self-efficacy; OR, odds ratio; CI, confidence interval; Profile 1, Low Flourishing-Low Meaning; Profile 2, Moderate Flourishing-Low Accomplishments; Profile 3, High Flourishing-Low Engagement.

## 7 Discussion

This multi-center study explored the heterogeneity of Flourishing levels in MHD patients, identifying three distinct latent profiles: Low Flourishing - Low Meaning (Profile 1), Moderate Flourishing - Low Accomplishments (Profile 2), and High Flourishing - Low Engagement (Profile 3). The findings highlight the importance of recognizing these distinct profiles when designing interventions and clinical care for MHD patients. Potential category ground predictors were also identified, namely social support, emotion regulation self-efficacy, coping styles, education, and marital status.

### 7.1 Latent profile characteristics of flourishing among MHD patients

Using LPA, this study identified three distinct flourishing profiles among MHD patients, highlighting the considerable variability in their flourishing levels. The Low Flourishing–Low Meaning group (20.48%) exhibits low flourishing overall, especially in the meaning dimension. These patients often experience physical distress from illness, coupled with financial and caregiving burdens on their families. As a result, they tend to have low self-worth and perceive their lives as lacking meaning (Zhu et al., 2024). The Moderate Flourishing–Low Accomplishment group (40.69%) scores lower in the accomplishment dimension. Because of the limitations imposed by hemodialysis, these patients frequently struggle to plan or set meaningful goals, adversely affecting their sense of achievement (Dąbrowska-Bender et al., 2018). The High Flourishing–Low Engagement group (38.83%) reports high overall flourishing but low engagement. Weakness and fatigue from illness likely reduce their daily activity levels and overall involvement in life (Jacobson et al., 2019).

### 7.2 Influences of demographic variables for the three profiles

Findings suggest that patients with only a high school education are more inclined to fall into Profiles 1 and 2. This may relate to a “sandwich effect”: high school–educated patients have higher self-expectations than those with lower education levels but lack the capacity or opportunities to fulfill these expectations. Consequently, the resulting “high expectations–low returns” paradox undermines their psychological adaptability, reducing happiness and a sense of meaning or achievement in life (Liu et al., 2019).

Unmarried and widowed patients are also more likely to be in Profiles 1 and 2, likely due to reduced emotional support, diminished social roles, heightened loneliness, and a weaker sense of life purpose. Unmarried patients lose the emotional regulation and support inherent in marriage, making them more vulnerable to low happiness and achievement under stress. Widowed individuals have lost an essential emotional anchor, and rebuilding life goals takes time, especially when coping with illness. By contrast, married patients often receive stable emotional support from their partners (Sułkowski et al., 2024). Divorced patients, although having lost their marital relationship, may have established new social networks, or found meaning through independent living. Interventions should therefore focus on strengthening social support, offering mental health services, and guiding these individuals in developing new interests and life goals.

### 7.3 Difference in the three profiles of flourishing in regulatory emotional self-efficacy

This study found that patients with low regulatory emotional self-efficacy (RES) tended to belong to Profiles 1 and 2, while those with high RES were more likely to be in Profile 3. This suggests that patients in Profiles 1 and 2 have insufficient confidence in regulating their emotions, while patients in Profile 3 are more confident in doing so. The concept of regulatory emotional self-efficacy was introduced by Italian psychologist Caprara and colleagues, referring to an individual’s confidence in their ability to regulate their emotional state (Caprara et al., 2008). Higher RES is associated with more effective emotion regulation strategies and greater subjective well-being (Wang et al., 2025). Such personal resources help individuals interact positively with the external environment and secure additional social resources. In contrast, patients with low RES are less able to manage the challenges of illness and struggle to access supportive social systems, leaving them feeling more confused and helpless.

### 7.4 Differences in the three profiles of flourishing in social support

Patients with low social support are more likely to fall into Profiles 1 and 2, while those with high social support are more prone to Profiles 2 and 3. This indicates that patients in profiles 1 and 2 perceive lower levels of social support, while those in profiles 2 and 3 experience higher levels of social support.

Social Support Resource Theory suggests (Hobfoll et al., 1990) that social support not only buffers stress but also fosters identity formation, community bonds, and a sense of belonging. Research has shown that social support directly impacts mental health and is a critical factor influencing psychological well-being. It provides emotional and practical assistance, boosts patients’ confidence to tackle future challenges, and alleviates psychological distress (Zhang and Dong, 2022). Conversely, insufficient support leads to unmet caregiving (Wang L. et al., 2023), emotional, and social needs, intensifying medical crises and weakening individuals’ self-worth and sense of life meaning (Cai et al., 2024).

### 7.5 Difference in the three profiles of flourishing in coping style

In this study, patients who frequently use passive coping are more likely to fall into Profiles 1 and 2, whereas those employing active coping are more likely to appear in Profiles 2 and 3. This phenomenon suggests that patients in profiles 1 and 2 are more inclined to adopt passive coping styles, while patients in profiles 2 and 3 are more likely to use active coping styles. Coping styles refer to the cognitive and behavioral strategies individuals employ when dealing with stressors. Positive coping involves proactive information-gathering and problem-solving strategies, which enhance confidence and perceived control over illness, thereby reducing psychological distress (Al Sharji et al., 2022). Passive coping, on the other hand, relies on avoidance or denial (Otto and Linden, 2020; Zhang et al., 2024), often leading to social withdrawal and diminished support. Over time, such withdrawal can intensify psychological problems such as anxiety, depression, and isolation (Liu et al., 2023).

## 8 Practical and research implications

Based on the research findings, the following recommendations are proposed. First, Interventions of Low Flourishing - Low Meaning should strengthen social support, improve regulatory emotional self-efficacy, and encourage active coping. Because the meaning dimension is low, therapies such as meaning-centered interventions (Eskigülek and Kav, 2024) or mindfulness training (Zimmermann et al., 2020) may help patients find and deepen their life purpose. Second, for Moderate Flourishing - Low Accomplishments, Priority should be given to boosting regulatory emotional self-efficacy, teaching emotional management skills, and guiding patients in maintaining emotional stability. Interventions might focus on helping patients set realistic fluid management or treatment goals, providing positive feedback and encouragement to enhance their sense of accomplishment. Last, Healthcare providers should focus on raising patients’ daily engagement levels for High Flourishing - Low Engagement, and this kind of patients should be encouraged to discover new hobbies or interests within the limits of their health status and practice being present in day-to-day activities.

## 9 Advantages and limitations

A key strength of this study is its groundbreaking exploration of latent flourishing profiles in individuals undergoing MHD. This study has several limitations. First, as a cross-sectional design, it cannot explore the causal relationships between the influencing factors and the three profiles. Future longitudinal or intervention studies are needed for validation. Second, the study participants were maintenance hemodialysis patients from Shanghai, which may result in sample homogeneity. The sample size should be expanded to enhance the representativeness in future studies.

## 10 Conclusion

This study identified three profiles of flourishing levels in MHD patients and their influencing factors through a cross-sectional survey. The results indicate that regulatory emotional self-efficacy, social support, and coping style impact the level of flourishing. The three profiles of flourishing revealed in this study shed light on the possibility of tailored intervention programs regarding Social Support Resource Theory and the flourishing profile of the MHD patients, thereby enhancing their long-term quality of life.

## Data Availability

The raw data supporting the conclusions of this article will be made available by the authors, without undue reservation.
